# Conjunctival lymphoma: case report

**DOI:** 10.22336/rjo.2025.69

**Published:** 2025

**Authors:** Andreea-Cristina Baltă, Mălina Adriana Mihai, Alexandru Mihai Ionescu, Mădălina Radu, Ioan Chițac, Gabriela Murgoi, Mihail Zemba

**Affiliations:** 1 Department of Ophthalmology, “Dr. Carol Davila” Central Military University Emergency Hospital, Bucharest, Romania; 2 Oncologic Institute of Bucharest, Bucharest, Romania; 3 “Carol Davila” University of Medicine and Pharmacy, Bucharest, Romania

**Keywords:** conjunctival lymphoma, extranodal marginal zone B-cell lymphoma, brachytherapy, OALs = ocular adnexal lymphomas, MALT = mucosa-associated lymphoid tissue, EMZL = extranodal marginal zone B-cell lymphoma, FL = folicular lymphoma, MCL = mantle cell lymphoma, TL = T-cell lymphoma

## Abstract

**Objective:**

To report the diagnosis and therapeutic approach in the case of a patient with conjunctival lymphoma.

**Case presentation:**

A 54-year-old caucasian female presented to the hospital with a painless conjunctival mass in the right eye of at least three months duration. The ophthalmological examination showed a “salmon patch” conjunctival lesion in the inferior fornix. An incisional biopsy of the conjunctival lesion was performed, and the histopathological examination confirmed the diagnosis of extranodal marginal zone B-cell conjunctival lymphoma. The CT scan showed no systemic involvement. It was decided to start radiotherapy with brachytherapy.

**Discussion:**

Conjunctival lymphoma is a rare ocular malignancy arising from polyclonal proliferation of lymphocytes. The most common subtype is extranodal marginal zone B-cell lymphoma (EMZL), followed by diffuse large B-cell lymphoma (DLBCL), follicular lymphoma (FL), mantle cell lymphoma (MCL), and T-cell lymphoma (TL). Clinical manifestations are non-specific; they usually present as a unilateral or bilateral painless, salmon-pink conjunctival lesion, often without systemic symptoms. Definite diagnosis requires conjunctival biopsy and relies on histopathology and immunohistochemistry, with molecular profiling playing an increasing role in risk stratification. Treatment strategies include: radiotherapy, immunotherapy (Interferon-α2b, Rituximab), chemotherapy, and antibiotherapy. Prognosis is generally favorable, with high survival rates, especially in localized cases.

**Conclusions:**

Conjunctival lymphoma can be easily overlooked during a routine examination; therefore, a thorough clinical evaluation, advanced imaging, histopathological, and immunohistochemical analysis are essential for appropriate management. Systemic staging and an interdisciplinary approach involving ophthalmology, medical oncology, and radiation oncology are necessary for optimal treatment planning and outcome.

## Introduction

Conjunctival lymphoma is a rare ocular malignancy arising from the clonal proliferation of lymphocytes within the conjunctiva, with a worldwide incidence of approximately 0.2 per 100000 [1,2]. Ocular adnexal lymphomas (OALs) constitute 8% of all extranodal lymphomas and 2% of non-Hodgkin lymphomas [3]. 25% to 30% of all OALs are located in the conjunctiva [4]. The incidence increased due to improved diagnostic technologies and increased reporting [1,5-8]. The most common subtype is extranodal marginal zone B-cell lymphoma (EMZL) of mucosa-associated lymphoid tissue (MALT), occurring in approximately 80% of cases, and is more frequent in females and in the 6th decade [9]. Other subtypes that can also be found are: folicular cell lymphoma (FL – 8%), diffuse large B-cell lymphoma (DLBCL – 3%), mantle cell lymphoma (MCL – 3%), and T-cell lymphoma (TL – 2%) [9]. EMZL and FL are generally characterized as low-grade forms of lymphoma. In contrast, MCL, DLCBL, and TL are considered high-grade lymphomas with a more aggressive growth pattern and potentially less favorable prognosis [1] (**Table 1**).

**Table 1:** Subtypes of conjunctival lymphoma

Histologic subtype	Histologic grade	Prevalence (%)	Typical Patient Demographics	Common Clinical Features	General Prognosis
EMZL/ MALT	Low-grade	68-81%	60-70 years, slight female predominance	Salmon-pink patch	Indolent
Follicular Lymphoma (FL)	Low-grade	8-16%	60-70 years, comparable sex distribution	Salmon-pink patch, multinodular appearance	Indolent
DLBCL	High-grade	3-4.6%	70s, male predominance	Grayish coloration	Aggressive
Mantle Cell Lymphoma (MCL)	High-grade	3-7%	70s, marked male predominance	May have bilateral manifestation	Poorer prognosis
T-cell/NK-cell	High-grade	2%	Insufficient data	May occur in the limbus	Poor prognosis

The etiology of conjunctival lymphoma remains unclear; however, several risk factors and potential etiological pathways have been identified in various studies. Chronic inflammation by prolonged antigen stimulation could lead to lymphoproliferations, which can potentially develop into conjunctival lymphoma and other ocular adnexal lymphomas. The predisposing risk factors incriminated in the pathogeny are: infectious agents (HIV, Hepatitis C virus, Epstein-Barr virus, Helicobacter pylori, Chlamydia psittaci, Borrelia burgdorferi), autoimmune conditions (Hashimoto thyroiditis, Graves disease, celiac disease, Sjögren syndrome, systemic lupus erythematosus, rheumatoid arthritis), benign lymphoid hyperplasia, genetic mutations (trisomy 3 and 18, 5q (ODZ2) and 9p ( JMJD2C), t(11;18)(q21;q21), t(14;18)(q32;q21), t(3;14) (p14.1;q32), and A20 inactivation (6q23 deletion)) and radiation exposure [1,2,7,9-16].

Clinical manifestations are non-specific; therefore, the diagnosis is relatively late. A unilateral or bilateral painless, pink, “salmon-patch”, sessile sub-epithelial mass with smooth surface and slow growing appears on the bulbar or fornical conjunctiva [9,17,18]. Less frequently, the lesion may be multinodular or follicular. Other signs and symptoms that may occur are: conjunctival hyperaemia, chemosis, dryness, epiphora, eye irritation, eyelid ptosis, diplopia, ocular motility disorders, or exophthalmos in case of significant orbital involvement [2,10]. Extra-ocular manifestations can show in the case of systemic dissemination.

The differential diagnosis encompasses inflammatory diseases, benign tumors, malignant tumors, and other conditions, such as chronic conjunctivitis, reactive lymphoid hyperplasia, conjunctival amyloidosis, atypical pterygium, episcleritis, pyogenic granuloma, amelanotic melanoma, and squamous cell carcinoma.

The initial evaluation of patients with conjunctival lymphoma requires careful ophthalmologic examination, a detailed patient history, and clinical examination. Biopsy for histopathological and cytological examination represents the gold standard for diagnosis; it can be incisional (for lesions larger than 10 mm, diffuse lesions, suspicion of systemic dissemination, bilateral lesions, and profound extension) or excisional (for lesions less than 10 mm, localised, mobile, and with well-defined limits) [1]. Histopathological analysis facilitates the identification of the type and grade of the lymphoma through the examination of lymphoid cell morphology and growth patterns. Hematoxilin-eosin staining shows a dense, monomorphic lymphoid infiltrate in the conjunctival stroma with the absence of epithelial disruption. Immunohistochemical stains with antibodies directed against CD3, CD5 (T-cell), CD20 (B-cell), CD79, Ki67 (proliferation index – tumor aggressiveness), kappa and lambda light chains, immunophenotyping, molecular genetic studies, and flow-citometry provide additional information for accurate subtyping [1,7,13,16,19].

High-Resolution Optical Coherence Tomography (HR-OCT) is a non-invasive imaging technique that aids in characterizing conjunctival lesions and monitoring disease resolution during treatment. Conjunctival lymphoma on HR-OCT may appear as hyporeflective, homogenous lesions with smooth borders and monomorphic dot-like infiltrates and a normal epithelium [9,20-23].

A systemic work-up should include: a complete blood count (CBC), lactate dehydrogenase (LDH) levels, serum protein electrophoresis, beta-2 microglobulin levels, and serum IgG4. A unilateral bone marrow aspirate and biopsy are often performed to establish the distant spread of the lymphoma [1,24].

Imaging techniques, such as computed tomography (CT), magnetic resonance imaging (MRI), and full-body positron emission tomography (PET-CT), play a crucial role in assessing both the local extent of the disease and systemic involvement [9,15,22,25,26].

Once a diagnosis of conjunctival lymphoma is confirmed, clinical staging of conjunctival lymphoma is determined by the Ann Arbor staging classification and the American Joint Committee on Cancer Tumor, Node, Metastasis (TNM) based staging system [7,27,28]. The Ann Arbor staging system is centered on clinical presentation, imaging, laboratory tests, and initial biopsy reports. Conversely, the TNM system evaluates the anatomic location of the tumor, tumor spread, lymph node involvement, and distant spread at the time of presentation and diagnosis.

The treatment approach for conjunctival lymphoma depends on the histological subtype, degree of dissemination, and patient-related factors such as age and comorbidities. External beam radiation therapy (EBRT) with lens shielding is the standard treatment for low-grade, isolated conjunctival lymphomas [25,29]. A dose range of 20-30 Gray fractionated in 15 sessions is usually prescribed, and the 5-year local control rate varies from 86% to 100% [30-35]. Side effects include: dry eye, eyelid irritations, mild conjunctivitis, ocular surface disease, periorbital tissue and palpebral disorders, cataract formation, keratopathy and radiation retinopathy, corneal ulceration, orbital fat atrophy [29,35-38]. Other treatment options include: brachytherapy, surgery, cyotherapy, immunotherapy with Interferon-alpha, Rituximab (anti-CD20 monoclonal antibody) or Daclizumab (anti-CD25 monoclonal antibody), radioimmunotherapy with Yttrium 90 ibritumomab tiuxetan, chemotherapy with a combined regimen (CVP = cyclophosphamide, vincristine, and prednisone or CHOP = cyclophosphamide, hydroxydaunorubicin, oncovin, and prednisone) [1,9,39-47]. Finally, antibiotic therapy with Doxycycline can be recommended when Chlamydia psittaci infection is associated with variable efficacy [1,6,19,25,48,49]. A “wait and watch” strategy has been proposed for elderly or frail patients with indolent tumors [50].

Overall, prognosis is generally favorable, with 90% of patients not experiencing progression or recurrence during a one-year follow-up period [51,52]. The survival rate varies between 50% and 94% based on the histological subtype, TNM stage at diagnosis, and patient age [1,9]. EMZL and FL are associated with a better outcome than LBCL, MCL, and T cell lymphoma [4,16,53]. Additionally, other elements have been identified as negative prognostic factors: bilateral lesions, age older than 60 years, elevated serum LDH levels, advanced disease stage, lymph nodes involvement, and presence of HCV infection [11,25,53,5,4-58]. BCL 6 (lymphoma-associated transcription factor), MUM1/IRF4 (multiple myeloma ocongene-1/interferon regulatory factor 4), and MIB1/Ki-67 (marker of cellular proliferation) are tumor markers associated with a higher risk for disseminated disease [56]. Systemic metastasis is rare; it occurs more commonly in DLBCL and MCL in the lymph nodes, bone marrow, salivary glands, liver, spleen, or lungs; 20% of cases evolve to systemic disease [1,28]. Long-term follow-up should be done at 3 months in the first year and every six months for an indefinite period.

## Case report

A 54-year-old Caucasian female presented to our clinic for ocular discomfort due to the appearance of a conjunctival mass in the right eye (RE), for at least three months, with a slow progressive evolution. The patient denied any significant family history of lymphoma and any current medications. Personal history was insignificant, with no documented infections, autoimmune diseases, reactive lymphoid hyperplasia, or radiation exposure.

**Fig. 1:**
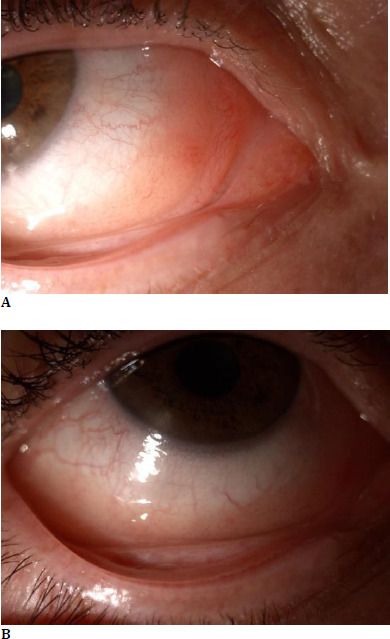
**A, B** Slit lamp image showing a conjunctival tumor with a salmon-pink appearance

On the ophthalmic exam, the patient’s best corrected visual acuity was 20/20 in both eyes with a normal intraocular pressure. Slit lamp examination of the anterior segment of the righ eye revealed: a conjunctival and subconjunctival salmon-pink infiltration of the intern canthus, inferior fornix and extern canthus, extending through the inferior tarsal conjunctiva, with a solid consistency, mobile over the underlying planes, with associated chemosis and increased superficial vascularization, smooth and transparent corneea, anterior chamber with normal depth, clear aquous humor, reactive pupil and transparent lens (**Fig. 1 A, B**). Slit lamp examination of the left eye and the fundus exam for both eyes were within normal limits. Clinical general exam was also within limits.

Based on the anamnesis, medical history, and clinical examination, the following diagnoses were established: right eye: conjunctival tumor formation at the level of the inferior fornix, and both eyes: hypermetropia and presbyopia.

Differential diagnosis included pingueculitis, chronic follicular conjunctivitis (small, round, pale, folicules on the palpebral conjunctiva often associated with chronic irritation and inflammation), reactive lymphoid hyperplasia, amelanotic melanoma, squamous cell carcinoma (nodular, vascularized, white-gray, irregular, gelatinous or papillomatous lesion, associated with HPV and exposure to UV), conjunctival amyloidosis (smooth, waxy, yellow-pink masses at the level of the lower fornix), and subconjunctival fat herniation (smooth, yellow mass protruding under the conjunctiva, associated with aging).

Reactive Lymphoid Hyperplasia (RLH) is a common entity in the differential diagnosis and can often be challenging to distinguish morphologically from malignant lymphoma. RLH frequently appears as bilateral and diffuse lesions, with self-limited progression and rare recurrence. On the other hand, conjunctival lymphoma presents as a usually unilateral and localised lesion with a slow-growing, progressive evolution and common recurrence. Biopsy with histopathological examination, immunohistochemistry, and molecular studies are required to differentiate these conditions. RLH shows polyclonal proliferation of lymphocytes with well-formed germinal centers in hematoxylin-eosin staining and a polyclonal kappa/lambda ratio (2/1) in immunohistochemistry. Conversely, in conjunctival lymphoma, hematoxylin-eosin staining reveals diffuse monoclonal proliferation without demarcated conjunctival follicles, and immunohistochemical examination indicates monoclonal kappa or lambda light chains dominance.

**Fig. 2:**
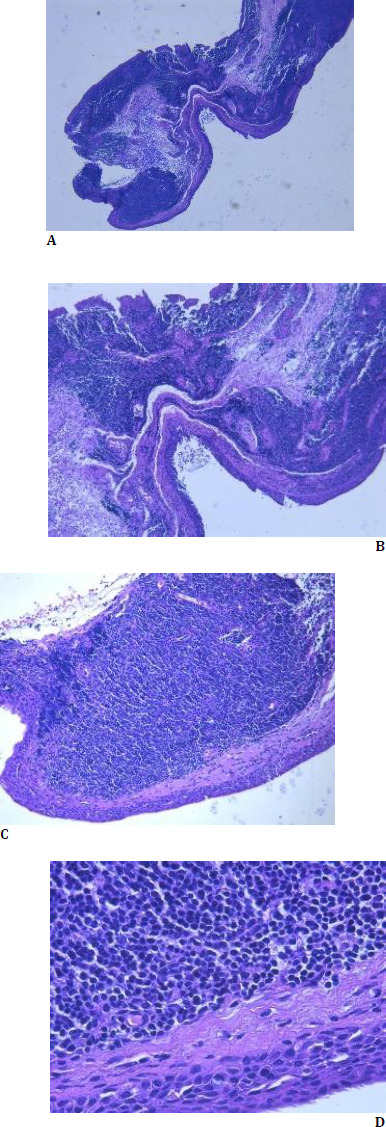
**A-D** Histological examination (H-E staining) shows diffuse lymphoid cell infiltration and a normal epithelium

Laboratory results and complete blood counts were within normal limits. As part of the incisional biopsy, a segment of conjunctival and subconjunctival tissue was excised from the level of the lower fornix of the right eye. First, the conjunctiva was pulled to observe where the most infiltrated area was, then xylin was injected under it. The conjunctival epithelium and subepithelial tissue from the area with a modified appearance were cut out and then sutured with 7.0 thread. Histopathological examination of the tissue sections with hematoxylin-eosin (H-E) staining revealed: diffuse subepithelial cellular proliferation, with lymphocytic-like cells (small-sized cells with hyperchromatic nuclei, with a relatively uniform appearance, without the formation of germinal centers, reduced cytoplasm) and a normal epithelium (**Fig. 2A-D**).

**Fig. 3:**
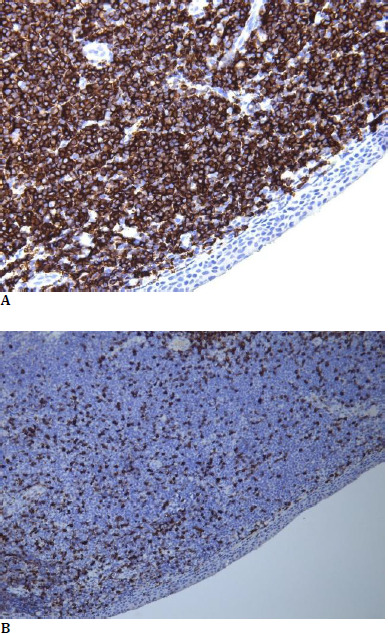
**A, B** Immunohistochemistry study for positive for CD20 (**A**), negative for CD5 (**B**)

The immunohistochemical examination identified tumor proliferation with small B-cells, positive for CD20 (positive marker in B-cells), negative for Cyclin D1, negative for CD5 (positive marker in T-cells), with a kappa/lambda ratio of 5/1 (monoclonal dominance of kappa light chains) (**Fig. 3A, B**).

The histopathological and immunohistochemical exam confirmed the diagnosis of extranodal marginal zone B-cell lymphoma.

After the biopsy, a hematological evaluation was requested, during which the patient underwent a CT scan of the orbit, thorax, abdomen, and pelvis, which identified several infracentrimetric retroperitoneal lymph nodes. In the hematology department, the result was considered inconclusive for systemic dissemination, and close follow-up was recommended. Following the mentioned investigations, a positive diagnosis of extranodal marginal zone B-cell conjunctival lymphoma, stage T1 N0 MO, and stage Ann Arbor 1 was made.

**Fig. 4:**
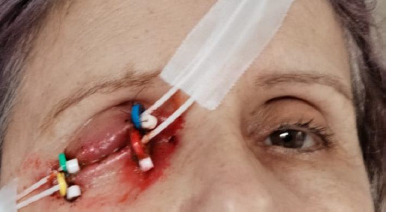
Brachytherapy

**Fig. 5:**
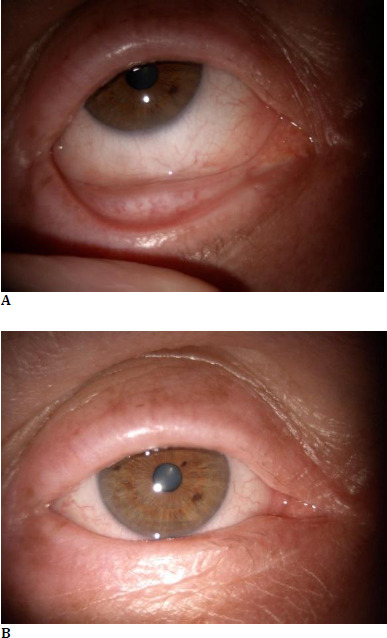
**A, B** Right eye, 10 months after treatment

At a new ophthalmological examination, a hematological consultation was recommended for a second opinion, and the patient was referred to the oncology institute. At the oncology institute, brachytherapy was instituted as a therapeutic measure, a type of radiotherapy that involves radioactive implants with strontium-90 or ruthenium-106 placed through the palpebral margin near the tumor formation (**Fig. 4**). During treatment, the patient received steroids, topical antibiotics, and artificial tears. After 10 months, the patient is currently under observation (**Fig. 5A, B**).

Following radiotherapy, the patient was recommended artificial tears and regular monitoring (**Table 2**).

**Table 2:** Long-term monitoring

Investigations	Frequency
Slit lamp examination + visual acuity + motility test + Schirmer’s test	3-6 months
HR-OCT of the anterior segment	6-12 months
Orbit MRI	6-12 months
PET-CT or CT	Anual
CBC, LDH, Beta2-microglobulin	6-12 months
Bone marrow biopsy	In case of suspected systemic involvement
Lesion biopsy	In case of suspected recurrence

## Discussion

The etiology and specific risk factors of conjunctival lymphoma remain not fully understood. Our patient presented none of the risk factors mentioned in the literature, such as autoimmune conditions (Sjogren’s syndrome, Hashimoto’s thyroiditis, rheumatoid arthritis), chronic inflammations, infections (Chlamydia psittaci, Helicobacter pylori, HVC), genetic predisposition, or radiation exposure [1,2,7,9,10].

Localised conjunctival lymphoma can be treated with EBRT, brachytherapy, immunotherapy, chemotherapy, cryotherapy, or surgery. EBRT and brachytherapy are the most widespread options, with numerous published studies reporting control rates between 86% and 100% [51]. Brachytherapy has fewer side effects, shorter treatment duration, and less radiation exposure compared to EBRT [59,60]. Brachytherapy can deliver high doses of radiation to target tissues while minimising collateral exposure to surrounding healthy tissues [60-62].

Local complications of conjunctival lymphoma are: foreign body sensation, irritation, dry eye syndrome, corneal infiltration, induced astigmatism, visual acuity decrease, proptosis, diplopia, compressive optic neuropathy, bacterial infections, corneal ulcer, and recurrence. Conjunctival lymphoma can be the first manifestation of systemic lymphoma. Systemic dissemination can cause lymphadenopathy, pancytopenia, anemia, and gastrointestinal tract diseases.

After brachytherapy, the patient presented loss of eyelashes, eyelid irritation, mild conjunctivitis, dry eye, and fatigue. Short-term effects of radiotherapy: conjunctivitis, keratitis, eyelid edema and erythema, meibomian gland dysfunction, loss of eyelashes, nausea, fatigue [1,17]. Long-term effects of radiotherapy: cataract, keratopathy, retinopathy, optic neuropathy, thinning and necrosis of the sclera [9,63].

In multiple studies, patients with unilateral conjunctival lymphoma, without systemic dissemination, without nodal involvement, at a low TNM stage at diagnosis, with a low-grade histology subtype, and aged less than 60 years had a better outcome [2,4,7,52,55]. The patient’s prognosis was favorable due to the following factors: histological subtype (EMZL), TNM stage at diagnosis (T1 N0 M0), patient age (54 years), presence of an isolated lesion without systemic dissemination, and early detection of the condition.

While radiotherapy remains the first-line treatment for localized disease, systemic involvement necessitates a more comprehensive approach that includes chemotherapy and immunotherapy. Although generally regarded as an indolent disease, conjunctival lymphoma has the potential for systemic progression, making long-term follow-up essential to detect recurrence or disseminated disease early. Recent advances in cytogenetics and molecular diagnosis have enhanced clinicians’ ability to predict future recurrence or systemic dissemination. Due to the significant rise in the incidence of ocular anexal lymphomas worldwide, future studies should enroll a higher number of patients and focus on further elucidating the roles of infectious agents, genetic alterations in the pathogenesis, prognostic markers, and treatment strategies to minimize long-term side effects.

The particularities of the case were: early onset in the 5th decade, early presentation in the ophthalmology department 3 months after the appearance of the lesion, and diagnosis of a potentially serious oncological condition following an ophthalmological examination for an apparently unimportant pathology.

## Conclusion

In conclusion, conjunctival lymphomas require a thorough patient history, an incisional biopsy with histopathological and immunohistochemical examination, a systemic work-up, and advanced imaging, such as CT or MRI, to confirm the diagnosis and establish the most effective treatment. While radiotherapy remains the first-line treatment for localized disease, systemic involvement necessitates a more comprehensive approach that includes chemotherapy and immunotherapy. Although generally regarded as an indolent disease, conjunctival lymphoma has the potential for systemic progression, making long-term follow-up essential to detect recurrence or disseminated disease early. Recent advances in cytogenetics and molecular diagnosis have enhanced clinicians’ ability to predict future recurrence or systemic dissemination. Due to the significant rise in the incidence of ocular anexal lymphomas worldwide, future studies should enroll a higher number of patients and focus on further elucidating the roles of infectious agents, genetic alterations in the pathogenesis, prognostic markers, and treatment strategies to minimize long-term side effects.
